# The Potential Protective Role of Naringenin against Dasatinib-Induced Hepatotoxicity

**DOI:** 10.3390/ph16070921

**Published:** 2023-06-23

**Authors:** Ahmed Z. Alanazi, Khalid Alhazzani, Salah Q. Alrewily, Khaldoon Aljerian, Mohammad M. Algahtani, Qamraa H. Alqahtani, Dhanush Haspula, Abdullah S. Alhamed, Mohammed Alqinyah, Mohammad Raish

**Affiliations:** 1 Department of Pharmacology and Toxicology, College of Pharmacy, King Saud University, Riyadh 12372, Saudi Arabia; kalhazzani@ksu.edu.sa (K.A.); 441106211@student.ksu.edu.sa (S.Q.A.); mohmalgahtani@ksu.edu.sa (M.M.A.); ghamad@ksu.edu.sa (Q.H.A.); asalhamed@ksu.edu.sa (A.S.A.); malqinyah@ksu.edu.sa (M.A.); 2 Department of Pathology, College of Medicine, King Saud University, Riyadh 12372, Saudi Arabia; kaljerian@ksu.edu.sa; 3 Molecular Signaling Section, Laboratory of Bioorganic Chemistry, National Institute of Diabetes and Digestive and Kidney Diseases, NIH, Bethesda, MD 20892, USA; dhanush.haspulagiridhar@nih.gov; 4 Department of Pharmaceutics, College of Pharmacy, King Saud University, Riyadh 12372, Saudi Arabia; mraish@ksu.edu.sa

**Keywords:** antioxidant, dasatinib, hepatotoxicity, inflammation, naringenin, protective effects

## Abstract

Dasatinib (DASA) is a novel tyrosine kinase inhibitor, approved for leukemia treatment. However, the long-term use of DASA induces several complications, especially liver damage. On the other hand, Naringenin (NGN) is a potent antioxidant and anti-inflammatory agent which is known to exert protective effects in several liver disease animal models. Yet, the effect of NGN on DASA-induced hepatotoxicity has not been examined. This study investigated the hepatoprotective effects of NGN against DASA-induced acute liver injury, using a mouse model. The mice were given NGN (50, 100, and 200 mg/kg po) or saline for 7 days, followed by DASA on the eighth day (25 mg/kg p.o.). DASA treatment alone was found to cause overexpression of proinflammatory cytokines, such as interleukin-10 (IL-10), tumor necrosis factor-alpha (TNF-α), and malonyl aldehyde (MDA), whereas attenuation of antioxidant genes including superoxide dismutase (SOD), catalase (CAT), glutathione S-transferase (GST), and glutathione peroxidase (GPx). Interestingly, a pretreatment with NGN + DASA resulted in minimizing the proinflammatory mediators and restoring the levels of antioxidant genes. In addition, there was evidence of necro-inflammatory changes in histopathological findings in the liver samples after DASA administration which remarkably reduced with NGN + DASA. Thus, this study revealed that NGN could minimize the hepatotoxicity induced by DASA by providing anti-inflammatory and antioxidant protection.

## 1. Introduction

Dasatinib (DASA) is a second-generation multi-tyrosine kinase inhibitor, that targets specifically BCR–ABL and sarcoma (Src) receptors, which have been implicated in leukemias. In fact, DASA has been approved for the treatments of Philadelphia-positive acute lymphocytic leukemia (ALL) and chronic myelogenous leukemia (CML) patients who are resistant or intolerant to imatinib. Evidence from preclinical studies revealed that DASA treatment showed the inhibition of tumor progression and metastasis in an intracranial CML mouse model where imatinib was ineffective. Clinically, DASA induced rapid hematologic and cytogenetic responses in patients with imatinib-resistant Philadelphia-positive ALL in a phase II study [1,2].

Despite several clinical and preclinical studies having confirmed the efficacy of DASA against various cancer cells, growing evidence indicates that DASA additionally attacks non-cancerous cells leading to unexpected side effects. These side effects include cytopenia, pleural effusions, and other moderate and controllable adverse actions such as fluid retention, dyspnea, gastrointestinal disorders, skin rash, headache, and fatigue [3]. In addition, several in vitro, in vivo, and clinical studies have reported that DASA causes hepatotoxicity. In preclinical in vitro studies, high levels of alanine aminotransferase (ALT) and lactate dehydrogenase (LDH) as well as the inhibition of rat primary hepatocytes cell viability were observed following treatment with DASA for 48 h [4]. Simultaneously, in vivo studies demonstrated that DASA had significantly increased levels of aspartate aminotransferase (AST), ALT, and LDH after treating rats for ten days accompanied by a significant reduction in the levels of antioxidants such as glutathione (GSH) and superoxide dismutase (SOD). In addition, DASA was proven to increase the levels of malondialdehyde (MDA), a lipid peroxidation indicator, nuclear factor erythroid 2-related factor 2 (Nrf2), and mitogen-activated protein kinases (MAPK) [5]. An elevation in circulating liver enzymes was additionally observed in clinical settings with DASA treatment in patients with CML [6]. The increased incidence of liver disease with DASA treatment when compared with nilotinib was reported in another clinical study, leading to an increased percentage of DASA discontinuation among chronic phase CML patients [6]. Apart from elevated ALT and AST, other adverse effects such as hypophosphatemia and hyperglycemia, were additionally reported in clinical settings [7].

Polyphenols are naturally occurring compounds present in plants, which are found in high levels in fruits and vegetables. Many studies have repeatedly shown a relationship between decreasing the risks of several illnesses such as cancer, cardiovascular diseases, diabetes mellitus, osteoporosis, and neurodegenerative diseases and the long-term consumption of a polyphenol-rich diet [8,9]. This inverse association between polyphenols and certain diseases indicates that polyphenols might possess a protective effect against many inflammatory diseases, via exerting antioxidant and anti-inflammatory effects [8,9].

Naringenin (NGN) is an essential compound that belongs to a subclass of flavonoids known as flavanones, which is considered a unique subclass of the polyphenol subclasses, and it is found in several citrus fruits: grapefruit, sour orange, tomato, and other fruits. Several biological activities have been attributed to NGN, such as antioxidant and anti-inflammatory effects. Several studies have shown that NGN plays a protective role against liver damage [10], in which NGN significantly reduced lipid peroxidation and restored antioxidant defense enzymes such as SOD, catalase, glutathione peroxidase (GPx), and glutathione S-transferase (GST) in a rat’s liver. Additionally, NGN treatment exhibited normal levels of serum albumin and total protein concentrations while reducing the concentration of liver collagen in a rat model of dimethylnitrosamine-induced hepatotoxicity [10]. Moreover, many studies have found that NGN prevented the increase in hepatic marker activities (AST, ALT, and alkaline phosphatase) in carbon tetrachloride-induced liver damage in rats [11,12]. NGN can relieve liver inflammation, as it acts as an anti-inflammatory agent by reducing or blocking nuclear factor-kappa B (NF-κB) which is responsible for the regulation of the proinflammatory protein expression of tumor necrosis factor alpha (TNF-α), interleukin-1β (IL-1β), and interleukin-6 (IL-6) [11]. Additionally, the degenerative effects of lead acetate on the liver were significantly decreased after the administration of NGN, suggesting potential hepatoprotective effects [13]. However, the hepatoprotective effect of polyphenols on the DASA-induced toxicities in the rodent model has not yet been investigated. Thus, this study was carried out to investigate the protective effects of NGN as a natural compound to alleviate DASA-induced hepatotoxicity.

## 2. Materials and Methods

### 2.1. Chemicals and Kits

DASA was obtained from LC Laboratories (Woburn, MA, USA), and NGN was purchased from Sigma Aldrich (St. Louis, MO, USA). Diagnostic kits for the measurement of ALT and AST activities, total protein, and serum parameters were purchased from RANDOX Laboratories (Ardmore, UK). ELISA kits for TNF-α, IL-6, MDA, GSH, and CAT were bought from Santa Cruz Biotechnology (Dallas, TX, USA).

### 2.2. Animals

Male Swiss albino mice (8–10 weeks old, weighing 25–30 g) were deployed in all the experiments. Mice were taken from the Experimental Animal Care Center of the College of Pharmacy, King Saud University (KSU), Riyadh, Saudi Arabia. Animals were housed under conventional laboratory conditions. The mice were fed a standard animal pellet diet and were allowed free access to water. All animal experiments were carried out under guidelines set by the Institutional Animal Care and Use Committee of KSU (IACUC reference number: KSU-SE-20-47).

### 2.3. Experimantal Design

#### 2.3.1. Determine DASA Tolerability and Survival Rates

Twenty-five male Swiss albino mice were randomly allocated into five groups, each group consisting of five mice. The groups were treated by oral gavage with increasing doses of DASA and assigned as follows: Group 1: the vehicle control group (0.5% carboxymethyl cellulose), Group 2: DASA dose of 25 mg/kg, Group 3: DASA dose of 50 mg/kg, Group 4: DASA dose of 100 mg/kg, and Group 5: DASA dose of 200 mg/kg. The survival rate of the mice was monitored over seven days, after being given different doses of DASA daily. The incidence of dead mice was counted every 24 h.

#### 2.3.2. Determine the Efficacy of Naringenin on DASA-Induced Liver Toxicity

**Figure 1:**
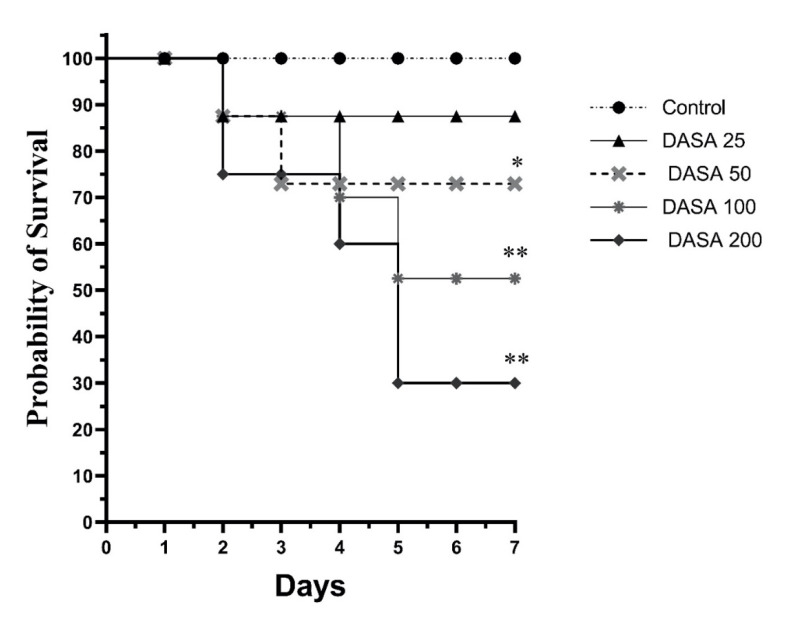
Effect of DASA on the survival rate. Mice received different doses of DASA (25 mg/kg, 50 mg/kg, 100 mg/kg, and 200 mg/kg; *n* = 5 per group). The survival rate was monitored for 7 days. The highest survival rate (87%) was recorded when mice received 25 mg/kg DASA, whereas the lowest survival rate (30%) was reported with 200 mg/kg DASA. * *p* < 0.05, ** *p* < 0.01 as compared to control. Kaplan–Meier curves and log-rank test were deployed to generate the survival rate curve.

Forty male Swiss albino mice were randomly divided into five groups, each group consisting of eight mice, as represented in Table 1. NGN and DASA were prepared and given daily by oral gavage. The dose of DASA (25 mg/kg) was selected based on the survival curve (Figure 1).

### 2.4. Samples Collection

At the end of the experimental period, all animals were fasted overnight and anesthetized with a ketamine (Hikma Pharmaceuticals, Amman, Jordan) plus xylazine (Laboratories Caliber, Barcelona, Spain) mixture. Blood samples were withdrawn from the heart and placed into clean tubes; next, the samples were separated by centrifugation at 3000 rpm (800× *g*) for 10 min and stored at −80 °C for further analysis. After this, ALT and AST levels were quantified by colorimetric methods (Linear Chemicals, Barcelona, Spain) [14].

The livers were harvested, and a portion of the liver of each experimental group was immersed and fixed in a 10% neutral formalin buffer (pH 7.4) for subsequent use in histopathological examinations for detecting changes in liver morphology and architecture. The other parts of the liver samples were immediately placed in liquid nitrogen for 1 min and then stored in the freezer at −80 °C until further analysis.

### 2.5. Tissue Analysis

The liver portions were homogenized in a cold physiological buffer of pH 7.4 (1:10, w/v), and the total protein concentrations in liver samples were measured according to a bicinchoninic acid (BCA) test (Santa Cruz, CA, USA). Thiobarbituric acid reactive substance (TBARS) and GSH levels were measured, using ELISA kits (Cayman Chemical Co., Ann Arbor, MI, USA). The hepatic levels of TNF-α, IL-6, and MDA were determined by following the ELISA techniques (R&D Systems Inc., Minneapolis, MN, USA). In post-mitochondria supernatants of liver samples, the enzymatic activities of CAT and GPX were measured using ELISA kits (R&D Systems Inc., Minneapolis, MN, USA).

### 2.6. Histological Evaluation

The mice’s livers were fixed in 10% formalin, dehydrated, and then embedded in paraffin wax. Using an automated microtome (Leica RM 2125 RM, Leica Microsystems, Nussloch, Germany), 4 µm sections were obtained and then mounted on glass slides. All slides were stained with hematoxylin and eosin (H&E) for examination under a Nikon Eclipse E600 microscope (magnification ×400), with a high-resolution digital camera [14]. Histopathological analysis and interpretation were performed by an experienced pathologist.

### 2.7. Gene Expression by Real-Time PCR

The total RNA from liver tissues was isolated using the TRIzol method [15]. The RNA quantity and quality were determined by NanoDrop 8000 (Thermo Fisher Scientific, Waltham, MA, USA), with an OD 260/280 range of ~2. Changes in the mRNA expression of IL-6 and TNF-α in response to DASA with or without NGN treatment were quantified by QuantStudio 6K Flex Real-Time PCR System, using SYBR Green Master Mix (MedChemExpress, Monmouth Junction, NJ, USA). The real-time PCR data were analyzed using the relative gene expression (i.e., 2^−ΔΔ^ CT) method. Briefly, the data were presented as a fold change in gene expression normalized to the endogenous reference gene (GAPDH) using the 2-delta–delta threshold cycle method (2^−ΔΔ^ CT method) [16].

### 2.8. Western Blotting for NF-kB

The total protein lysate was prepared by homogenizing 50 mg of tissue in an ice-cold box for 30 s on and 30 s off, 2 or 3 times in a 400 µL RIPA lysis buffer mixed with 4 µL of protease inhibitor (ThermoFisher, Waltham, MA, USA). Protein lysates were then incubated for 15 min, followed by centrifuging at 16,000 rpm for 20 min at 4 °C. After that, the supernatants were collected and stored at −20 °C. The protein concentration for each protein sample was quantified, using the bicinchoninic acid (BCA) test (Santa Cruz Biotechnology, Dallas, TX, USA).

Protein samples were mixed with distilled water and 2× Laemmli sample buffer to create the total protein lysates (Santa Cruz, CA, USA). After that, samples were denatured by heating at 50 °C for 5 min. Protein was resolved on 10% SDS-polyacrylamide gel, and the gels were then electrophoresed at 110 volts for 80 min. Approximately 20 g of whole protein lysate for each sample was loaded into the gel. Following gel electrophoresis, proteins were transferred into nitrocellulose membranes (Santa Cruz Biotechnology, Santa Cruz, CA, USA) using Transfer-Blot SD (Bio-Rad, Hercules, CA, USA). The membranes were then blocked with bovine serum albumin (BSA) to minimize nonspecific binding and then washed with TBST 3 times for 5 min each. The membranes were then incubated with the primary antibody, NF-κB (Santa Cruz Biotechnology, Santa Cruz, CA, USA) overnight at 4°C. The next day, the membranes were washed 3 times for 5 min each with TBST, then incubated with 7 mL of BSA containing a 1.5 μL anti-mice secondary antibody for 1 h at room temperature. Subsequently, the membrane was washed 3 times in TBST for 5 min each, then imaged using the Gene Gnome XRQ system. As a loading control, GAPDH (Bio-Rad Laboratories, Hercules, CA, USA) was used in our analysis. The imaged bands were analysed using the ImageJ software (National Institutes of Health, Bethesda, MD, USA).

### 2.9. Statistical Analyses

Statistical analyses were performed using GraphPad Prism (version 8) software. All data were expressed as mean ± standard deviation (SD). The statistical significance of differences was calculated using a one-way analysis of variance (ANOVA), followed by a post hoc Tukey–Kramer multiple comparison test. Statistically significant differences among the various treatment groups were assumed when *p* <0.05.

## 3. Results

### 3.1. Effect of DASA Doses on Survival Rate

The survival rates of the different DASA doses were compared, as indicated in Figure 1. At the end of treatment, no mortality was recorded in the control group (vehicle-treated group). Indeed, the highest survival rate after the control was noticed when mice were treated with DASA at a dose of 25 mg/kg (87%). On the contrary, mice treated with 200 mg/kg of DASA had the lowest survival rate (30%), followed by 100 mg/kg of DASA (52%). Furthermore, the rate of survival was increasing as the doses of DASA reduced where the DASA at 50 mg/kg resulted in a survival rate of 72.9%. Therefore, the dose of 25 mg/kg DASA was selected for further analysis due to its tolerability, highest survival rate, and lowest mortality rate.

### 3.2. Effect of NGN ± DASA on Mice’s Bodyweights

**Figure 2:**
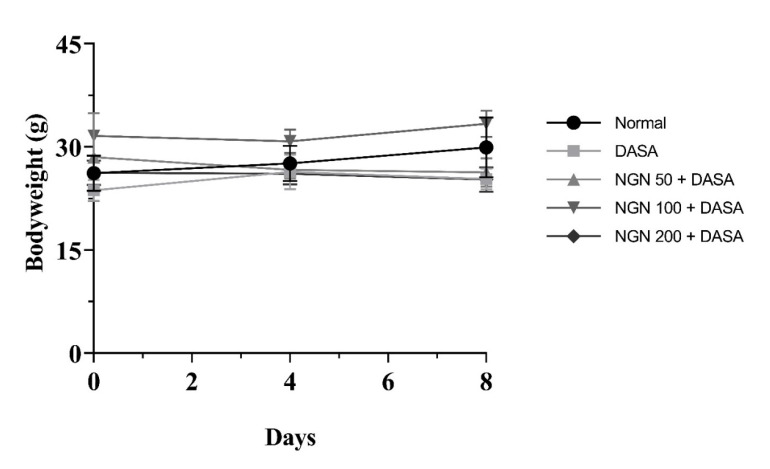
Effect of NGN ± DASA on mice bodyweights. Mice received different doses of NGN orally (50 mg/kg, 100 mg/kg, and 200 mg/kg) for 7 days, followed by a single dose of DASA (25 mg/kg) on the 8th day. During the experiment, the body weight of mice was measured every four days. Each experimental group contained eight mice. No significant change in bodyweight was observed at the 8th day among various treatment groups, as compared to the control (*p* > 0.05). Data are plotted as means ± SD; *n* = 8.

Changes in bodyweight were monitored, as a presumptive sign of the tolerability of NGN treatment. Therefore, we recorded the weight of the mice at the beginning of the daily treatment with different NGN doses (50 mg/kg, 100 mg/kg, and 200 mg/kg) for 7 days followed by a single dose of DASA (25 mg/kg) on the 8th day (Figure 2). Results showed that the mice’s body weights were not significantly affected by different concentrations of NGN in all the groups, compared to the control in the experiment (Figure 2).

### 3.3. Effect of NGN ± DASA on Liver Enzymes

**Figure 3:**
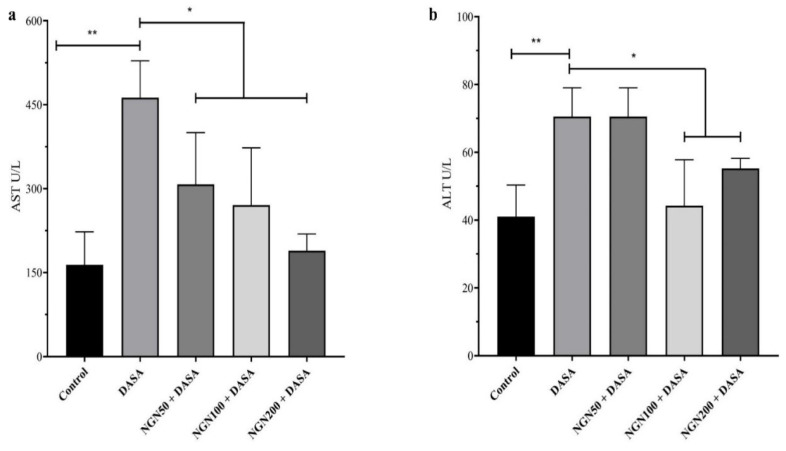
Effect of NGN ± DASA on liver enzymes. Mice received daily pretreatment with NGN (50 mg/kg po), NGN (100 mg/kg po), or NGN (200 mg/kg po) for 7 days, followed by a single dose of DASA (25 mg/kg, po) on the 8th day. (**a**) Serum AST levels in various treatment groups. (**b**) Serum ALT levels in various treatment groups. Data are presented as means ± SD. * *p* < 0.05, ** *p* < 0.01, *n* = 5.

The serum levels of AST and ALT were measured as biomarkers associated with liver injury and damage. The results showed the levels of AST and ALT were significantly (*p* < 0.01) elevated after DASA treatment, compared with the control group (Figure 3). Daily pretreatment with NGN for 7 days, followed by a single dose of DASA significantly reduced the serum levels of AST and ALT as compared to DASA (*p*< 0.05). Furthermore, increased doses of NGN resulted in a statistical decrease in AST levels compared with the DASA group (Figure 3a). Nevertheless, ALT levels showed a significant decrease only in the 100 mg/kg and 200 mg/kg NGN groups in comparison with the 50 mg/kg NGN group and DASA group (Figure 3b).

### 3.4. Effect of NGN ± DASA on Liver Histopathology

**Figure 4:**
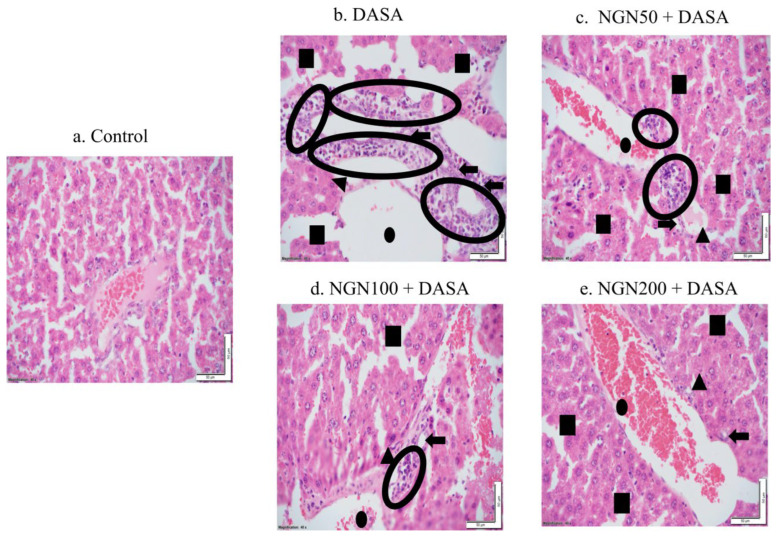
Histomicrographs of liver sections obtained from various treatment groups. Control group showed no sign of inflammation, whereas DASA groups showed signs of acute inflammation, severe neutrophils, and lymphocytes infiltration. Group NGN 50 + DASA exhibited moderate inflammation, moderate neutrophils, and lymphocytes infiltration however to a lesser degree compared with the DASA group. Mild inflammation with a remarkably reduced amount of inflammation and damage was noticed in NGN 100 + DASA, compared to NGN 50 + DASA group and DASA group. NGN 200 + DASA group exhibited no signs of liver tissue inflammation and damage, despite being administered with DASA. Dot: portal venule; arrow head: arteriole; arrow: branch of bile duct; and square: hepatocytes.

To investigate whether DASA induced changes in liver morphology and architecture, liver samples from different treatment groups were stained with hematoxylin and eosin to conduct histopathological analysis and a comparison between the control and DASA (25 mg/kg) ± different doses of NGNs (50 mg/kg, 100 mg/kg, and 200 mg/kg). The control group exhibited a normal morphology and architecture of liver tissue, with no sign of inflammation (Figure 4), whereas the liver samples of DASA-treated mice showed acute inflammation, severe neutrophils, lymphocytes, and plasma cell infiltration compared with the control group (Figure 4). Nevertheless, Figure 4c–e shows the efficacy of NGN in different doses (50 mg/kg, 100 mg/kg, and 200 mg/kg) in reducing liver damage induced by DASA, in comparison with DASA alone. We noticed that the gradual increase of the NGN dose from 50 mg/kg to 100 mg/kg and reaching 200 mg/kg resulted in better amelioration and recovery of the liver tissues from the deleterious effects associated with DASA administration, in the form of fibrosis and necrosis.

### 3.5. Effect of NGN ± DASA on Gene Expression of TNF-α and IL-10

**Figure 5:**
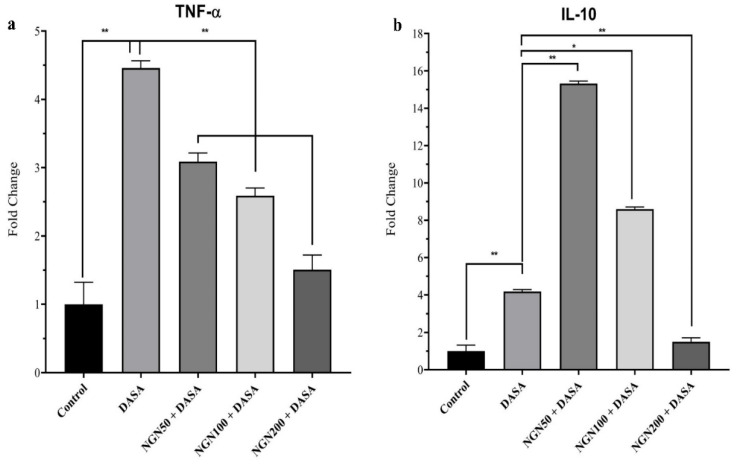
Effect of NGN ± DASA on gene expression of TNF-α and IL-10. The mRNA expression levels of proinflammatory cytokines (**a**) TNF-α and (**b**) IL-10 were measured by RT-PCR in various treatments. Data are presented as means ± SD. * *p* < 0.05, ** *p* < 0.01, *n* = 5.

We sought to compare the differential gene expression of proinflammatory cytokines, such as TNF-α and IL-10. Our results showed the DASA group significantly increased (*p* < 0.01) the gene expression of TNF-α and IL-10, as compared to the control group (Figure 5). The gene expression of TNF-α, as a proinflammatory cytokine gene, was significantly reduced by different doses of NGN + DASA compared with DASA (Figure 5a). Surprisingly, the expression of the IL-10 gene was elevated in the NGN (50 mg/kg and 100 mg/kg) + DASA groups compared with the DASA group (Figure 5b). On the other hand, the NGN (200 mg/kg) + DASA groups significantly reduced the IL-10 expression compared to the DASA group (Figure 5b).

### 3.6. Effect of NGN ± DASA on Antioxidant Genes (CAT, SOD2, GST, and GPX)

**Figure 6:**
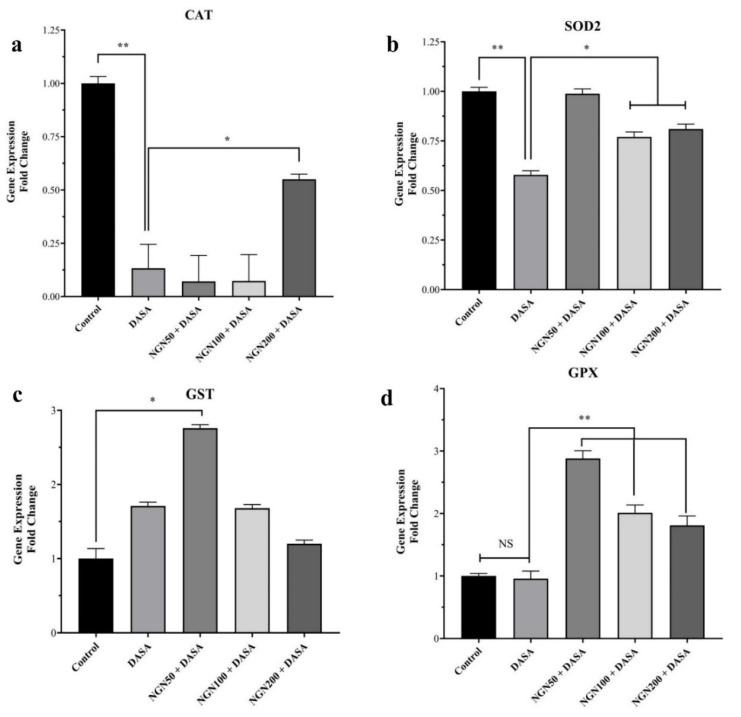
Effect of NGN ± DASA on antioxidant genes. The mRNA expression levels of antioxidant genes (**a**) CAT, (**b**) SOD2, (**c**) GST, and (**d**) GPX were measured by RT-PCR in various treatment groups. Data are presented as means ± SD. NS means no significant changes were observed (*p* > 0.05). * *p* < 0.05, ** *p* < 0.01, *n* = 5.

The antioxidant gene expressions of CAT, SOD2, GST, and GPX were measured in liver tissue lysate obtained from various treatment groups. As shown in Figure 6a, the expression of CAT statistically decreased in the DASA group, even when it was pretreated with NGN (50 and 100 mg/kg). NGN 200 mg/kg + DASA showed a slight elevation in CAT expression (Figure 6a). The expression of SOD2 was significantly elevated in all NGN pretreated groups, compared to the DASA group (Figure 6b). Regarding GST, the DASA group showed a high elevation in its expression compared with the control group. Furthermore, the NGN 50 mg group represented the most significant increase in GST expression compared to DASA and other NGN-treated groups (Figure 6c). Lastly, GPX expression was significantly elevated in all NGN pretreated groups, with the dose of 50 mg having the most profound effect (Figure 6d).

### 3.7. Effect of NGN ± DASA on Tissue Concentrations of TNFα, IL-6, CAT, GSH, and MDA Levels

**Figure 7:**
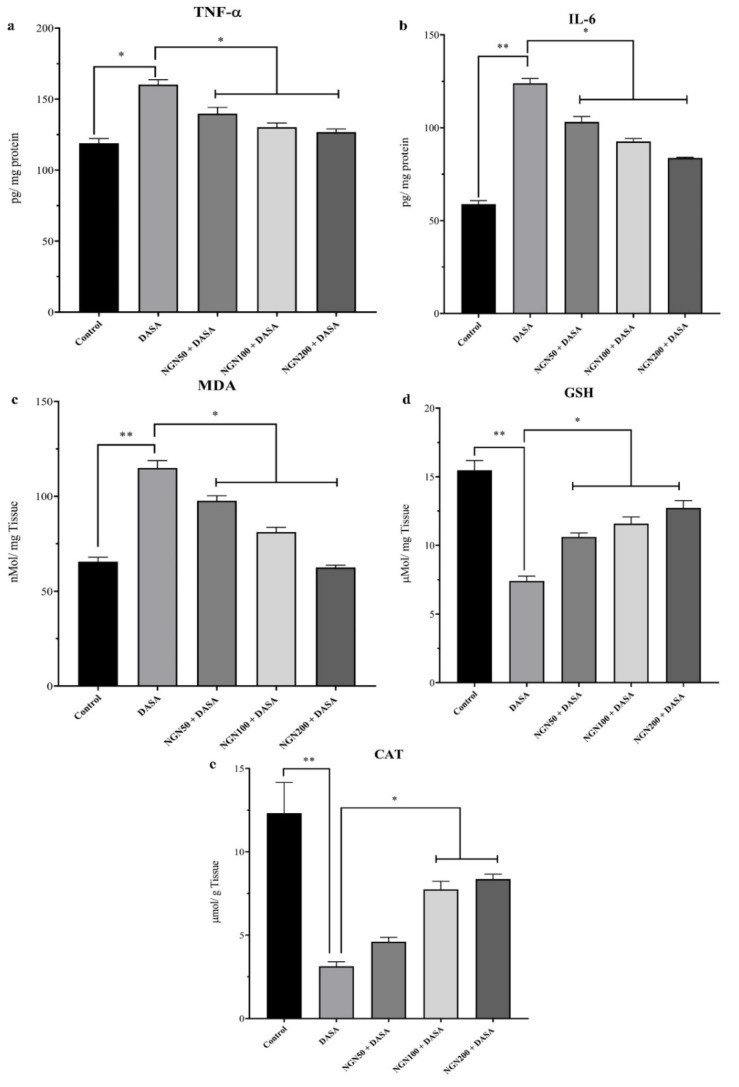
Effect of NGN ± DASA on proinflammatory cytokines and oxidative stress/antioxidant proteins. The liver tissue protein levels of proinflammatory cytokines (**a**) TNF-α and (**b**) IL-6, and (**c**) the tissue levels of MDA as an indicator of oxidative stress, as well as antioxidant proteins (**d**) GSH and (**e**) CAT. Protein levels were measured for various treatment groups using ELISA. * *p* < 0.05, ** *p* < 0.01.

Comparisons of the protein concentrations of proinflammatory cytokines (TNF-α and IL-6), antioxidant proteins (CAT and GSH), and MDA as markers of oxidative stress were performed using the ELISA technique. As shown in Figure 7, the levels of TNF-α, IL-6, and MDA were significantly decreased in the NGN 50 mg/kg group compared with the DASA group. However, increasing the doses of NGN had a slight gradual effect on such parameters (Figure 7a–c). In addition, significant elevations in the concentrations of CAT and GSH were observed in the NGN 50 mg group compared with the DASA group, and this effect was directly proportional to the increasing dose of NGN (Figure 7d,e).

### 3.8. Effect of NGN ± DASA on the Expression of NF-κB Protein

**Figure 8:**
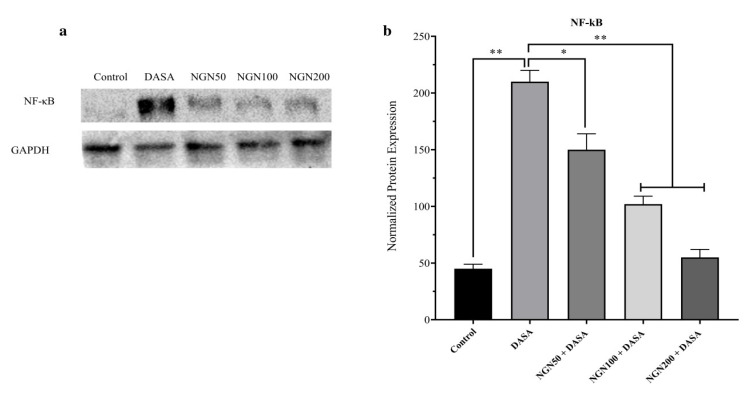
Effect of NGN ± DASA on the expression of NF-κB protein. (**a**) The western blotting analysis revealed an increase in NF-κB protein expression compared to the control, and this effect was reduced gradually with an increasing dose of NGN. (**b**) Densitometry analysis displayed the relative changes in the levels of NF-κB. Data are presented as means ± SD. * *p* < 0.05, ** *p* < 0.01.

The NF- κB expression in the various groups of mice was determined via the western blotting technique. As shown in Figure 8a, DASA treatment alone resulted in a significant increase in the expression of NF-κB compared to the control. Interestingly, the expression of NF-κB was significantly reduced after NGN treatment. Furthermore, the reduction in the expression of NF-κB was directly proportional to the doses of NGN. Figure 8b shows the protein expression of NF-κB, normalized to the control (GAPDH) in all the treatment groups.

## 4. Discussion

Leukemia is considered a potentially fatal malignant disorder of the blood and bone marrow. Epidemiologically, the incidence of leukemia, especially AML and CLL types, showed a remarkable elevation worldwide in the last three decades. As a result, it has become a significant global public health issue [17]. The aggressiveness of leukemia is directly proportional to age, and the treatment strategies are becoming less tolerable [18]. Despite its undesirable adverse effects and resistance, chemotherapy has been considered the first choice in the treatment of various types of leukemia for many years. With the progress of science, targeted cancer therapy, additionally called the “magic bullet concept”, is becoming an ideal alternative to chemotherapeutic agents due to its efficacy in target leukemic cells with fewer side effects in other healthy tissues compared to traditional chemotherapy.

An example of targeted therapy is tyrosine kinase inhibitors (TKIs). These drugs are highly efficacious in the treatment of leukemia, especially in CML and Ph+ ALL patients. Subsequently, TKIs are becoming a targeted approach and serving as an effective therapeutic plan for those patients with a variety of drug generation options [19,20]. DASA, as a member of the second-generation TKIs, was approved for the treatment of all CMLs and Ph + ALL patients, especially in cases of imatinib resistance. Despite its advantages in leukemia treatment, DASA has been linked to hepatotoxicity in clinical trials. As a result, liver dysfunction during treatment with DASA is receiving a lot of attention, particularly in long-term use. Thus, identifying new defense strategies against DASA-induced liver damage could help improve the clinical efficacy and safety of DASA.

NGN is a citrus flavonoid that belongs to the polyphenol group. This phytochemical has been associated with many biological activities, including antibacterial, antiviral, antioxidant, anticancer, anti-inflammatory, antiadipogenic, and cardioprotective properties, which have been reported in several in vitro and in vivo studies [21,22,23]. Nevertheless, there have additionally been several clinical trials, with the major focus being on NGN bioavailability and cardioprotective effects [10].

This current study was conducted to investigate whether NGN exerts a protective role against DASA-induced hepatotoxicity, determine the downstream effect of DASA on inflammatory and oxidation modulators, and discern the role of the NF-κB pathway in hepatocyte damage. This additionally includes examining the role of NGN in inducing anti-inflammatory and antioxidant activity in liver toxicity conditions. To test our hypothesis, we used DASA as an anticancer agent to induce hepatotoxicity and NGN as a hepatoprotective agent and evaluated the effect of the treatment combination on inflammation and oxidation modulation. To achieve our aims, we investigated the effects of therapy with different doses of DASA on survival rate. We found that DASA at 25 mg/kg has the highest survival rate with low mortality, compared to higher doses (50 mg/kg, 100 mg/kg, and 200 mg/kg). Next, we tested the pretreatment of different doses of NGN + DASA on mice’s body weight and liver enzymes (AST and ALT), conducted a histopathological examination, and calculated the changes in TNF-α and IL-10, SOD2, GST, CAT, and GPX gene expression levels. Additionally, we performed immunoblotting for the NF-κB protein expression as well as investigating the levels of TNF-α, IL-6, MDA, CAT, and GSH protein expression in liver lysates.

First, we estimated the survival rate to investigate the severity of DASA toxicity on hepatocytes as performed in previous studies [4,24,25]. Mice received doses of DASA (25 mg/kg, po; 50 mg/kg, po; 100 mg/kg, po; and 200 mg/kg, po). At the end of the experiment, the survival rate had reduced to 30% in the DASA 200 mg/kg group, 52% in DASA 100 mg/kg, and 72.9% in DASA 50 mg/kg groups. The DASA 25 mg/kg group resulted in the highest survival rate at 87%, compared to the control where no deaths occurred in the mice. Obviously, the rate of mortality was directly proportional to increasing the doses of DASA in our results which demonstrates the severity of DASA on liver toxicity along with increasing doses. DASA at 25 mg/kg was employed to induce hepatotoxicity, as a lower mortality was observed with this dose compared to the higher doses (50 mg/kg, 100 mg/kg, and 200 mg/kg). The bodyweights of the mice were monitored during the course of the study. No statistical significance was observed during therapy in all NGN groups, compared to the control in the experiment, indicating a high degree of tolerability. Furthermore, the measurement of liver enzymes (ALT and AST) to measure the degree and severity of liver damage showed a statistical increase in the DASA group compared with the control group. These results are compatible with other, previous studies [4,5]. On the other hand, we noted a significant decrease in AST and ALT levels after NGN pretreatment. High doses of NGN resulted in a significant decrease in AST levels, compared with the DASA group, whereas ALT levels showed a significant reduction only with the 100 mg/kg and 200 mg/kg NGN doses. The protective effect of NGN on the liver, which was represented by the reduced levels of ALT and AST, comes in parallel with other previous works which showed that NGN has a hepatoprotective effect against many drugs that induce hepatotoxicity [26,27,28].

Histopathology examinations revealed increased inflammation and infiltration of neutrophils, lymphocytes, and plasma cells, as well as severe interface hepatitis and bridging necrosis in the DASA group compared to the control. The administration of different doses of NGN significantly reduced the severity of inflammation and necrosis in liver tissues. Furthermore, in the NGN 200 mg/kg + DASA group, there was a complete recovery and normal appearance of liver with no sign of inflammation. Our histopathological findings are compatible with several reports indicating the efficacy of NGN against the toxicity of several drugs on hepatocytes [13,27,29].

One of our goals was to investigate the effects of the various treatments on NF-κB (a well-known transcriptional regulator of proinflammatory cytokines) expression in hepatic cells in an attempt to study the anti-inflammatory and antioxidant activities of NGN. We found an increased expression of NF-κB in the DASA group compared to the control, which indicated the activation of the inflammatory pathway in hepatic cells. Significantly, all NGN groups appeared to reduce the proinflammatory pathway via minimizing the level of NF-κB expression. In addition, it is possible to improve hepatic cell survival via a targeted pathway disruption of NF-κB. Indeed, inflammation is associated with hepatotoxicity in rodents through increasing the level of inflammation indicators, such as cytokines and C-reactive protein in the liver [30,31,32]. Moreover, several inflammatory mediators may be involved in the mechanism of the defective liver, such as malondialdehyde (MDA), nuclear factor erythroid 2-related factor 2 (Nrf2), and mitogen-activated protein kinases (MAPK) [4,5]. While there has been no suggestion of specific molecular processes associated with hepatic cell impairment induced by DASA through the NF-κB pathway, this study examined the outcomes of mice exposed to a certain concentration of DASA with/without NGN on hepatocytes.

We additionally examined changes in the gene expression of the proinflammatory cytokines genes (TNF-α and IL-10) as well as antioxidant genes (CAT, SOD2, GST, and GPX) to evaluate the effect of NGN against DASA on hepatocytes. Although TNF-α was highly expressed in the DASA group compared to the control, NGN was able to significantly reduce its expression. Conversely, the expression of IL-10 was reduced in the DASA group and elevated in all the NGN groups. In fact, IL-10 is a key cytokine that can be produced by numerous cell types within the liver including hepatocytes and Kupffer cells [33,34]. This cytokine has potent anti-inflammatory properties, and many studies indicated that IL-10 can modulate the inflammatory response and limit the liver damage in various animal models with liver injury [35,36,37,38]. There are several mechanisms that could provide explanations for the protective role of IL-10 in liver damage [39]. These mechanisms include the ability of IL-10 to inhibit the release of TNF-α by Kupffer cells, since the activation of these cells has been confirmed to play a deleterious role in various liver disease models [39]. In addition, this cytokine was able to downregulate the adhesion molecules which resulted in the inhibition of the adherence of neutrophils to the endothelium [39]. Moreover, growing evidence revealed that IL-10 can inhibit the toxic mediators that are induced by neutrophils. In this current study, we showed that IL-10 levels were increased significantly in the NGN 50 + DASA and NGN 100 + DASA treatment groups which can be considered one of the potential underling mechanisms by which NGN could protect the liver from damage by DASA. Moreover, the administration of NGN remarkably increased the expression of all antioxidant genes except CAT. These findings are correlated with several reports about NGN against several drugs which induced hepatotoxicity [13,27,29]. In addition, our results showed significant antioxidant protective effects of NGN by the elevation of antioxidant proteins (CAT and GSH) and anti-inflammatory action through a significant reduction in pro-inflammatory proteins (TNF-α and IL-6) and the MDA protein.

In conclusion, this study examined the role of NGN as a potential hepatoprotective therapy against DASA-induced liver toxicity. High doses of DASA had a detrimental effect in inducing mortality and decreasing the survival rate in our study. In addition, there was significant evidence in the reduction of elevated liver enzymes ALT and AST as well as an improvement in liver tissue damage after NGN intake. DASA treatment was found to cause an overexpression of proinflammatory mediators, including TNF-α, MDA, IL-10, and IL-6 as well as the attenuation of antioxidant genes CAT, SOD, GPx, and GST. However, NGN treatment significantly reversed the activities of inflammatory mediators and restored the levels of antioxidant proteins and genes. Moreover, NGN was able to minimize the upregulation of NF-κB induced by DASA which would confirm its crucial role against DASA-induced hepatotoxicity. Further studies will be required to investigate the underlying molecular mechanisms by which the NGN showed its protective effects.

## Figures and Tables

**Table 1:** Study design for determining the efficacy of different doses of NGN on DASA-induced liver toxicity.

Groups	*n*	Treatment Plan
Control	8	Received the vehicle (0.5% carboxymethyl cellulose) by oral gavage daily for 8 days.
DASA	8	Received the vehicle (0.5% carboxymethyl cellulose) daily for 7 days and subsequently treated with DASA (25 mg/kg) on the 8th day.
NGN50 + DASA	8	Received NGN (50 mg/kg, solubilized in 0.5% carboxymethyl cellulose) daily for 7 days and were subsequently treated with DASA (25 mg/kg) on the 8th day.
NGN100 + DASA	8	Received NGN (100 mg/kg, solubilized in 0.5% carboxymethyl cellulose) daily for 7 days and were subsequently treated with DASA (25 mg/kg) on the 8th day.
NGN200 + DASA	8	Received NGN (200 mg/kg, solubilized in 0.5% carboxymethyl cellulose) daily for 7 days and were subsequently treated with DASA (25 mg/kg) on the 8th day.

## Data Availability

The data used to support the findings of this study are included within the article.
